# Supplementary material for: Characterization of Prophages and Their Genetic Cargo in Clinical M. abscessus Isolates
Source: Microorganisms. 2025 Aug 30;13(9):2028. doi: 10.3390/microorganisms13092028 (PMC12472911; doi:10.3390/microorganisms13092028)
Supplement: Supplementary file 1 [file microorganisms-13-02028-s001.zip › microorganisms-3801043-supplementary.pdf]

Supplementary Materials

Supplementary Table S1: Basic Information of Genomic Sequencing of *M. abscessus*.

	Mean ± SD	Median	95% CI
**Total Scaffold Count**	145.8 ± 299.2	99	66.4–225.2
**Total Base Count (bp)**	5,095,655 ± 312,714	5,072,340	5,012,681–5,178,629
**Scaffold N50 (bp)**	170,886 ± 73,870	157,293	151,286–190,486
**Scaffold N90 (bp)**	49,556 ± 23,763	48,750	43,251–55,861
**GC Content (%)**	64.2 ± 0.57	64.1	64.1–64.4
**N Base Content (%)**	0.01 ± 0.03	0.005	0.00–0.02
**Total Contig Count**	213.1 ± 453.1	142	92.8–333.3
**Sequencing Depth**	263.7 ± 29.2	261.6	255.9–271.4
**Sequencing Completeness (%)**	96.2 ± 0.9	96.7	96.0–96.4

Supplementary Table S2: Genomic Annotation and Composition Analysis of *M. abscessus*

	Mean ± SD	Median	95% CI
**Total Annotated Genes**	5,110 ± 568	5,062	4,960–5,261
**Total Annotated Gene Length (bp)**	4,713,071 ± 274,907	4,685,862	4,640,128–4,786,014
**Average Gene Length (bp)**	925.6 ± 30.65	929	917.4–933.7
**GC Content in Gene Regions (%)**	64.6 ± 0.60	64.5	64.4–64.7
**Gene Coverage (%)**	92.5 ± 0.33	92.5	92.4–92.6

**Integrated Gene Region Length (bp)**	382,584 ± 39,617	384,103	372,072–393,096
**GC Content in Integrated Regions (%)**	59.9 ± 0.71	59.9	59.7–60.1
**Integrated Region Coverage (%)**	7.5 ± 0.33	7.54	7.41–7.58
**Virulence Factor Count**	354.0 ± 28.7	350	346.4–361.6
**Resistance Gene Count**	152.9 ± 18.1	151	148.1–157.7
**Secretion System Count**	14.2 ± 0.6	14	14.0–14.4
**Sec-SRP Secretion Systems**	11.2 ± 0.5	11	11.1–11.3
**Tat Secretion Systems**	3.0 ± 0.1	3	3.0–3.1
**Transport Protein Count**	656.2 ± 70.0	646	637.7–674.8
**Transmembrane Protein Count**	1,134 ± 123	1,120	1,102–1,167

Supplementary Table S3: Average Count of Detected Virulence Factors and Resistance Genes in *M. abscessus.*

	**Mean ± SD**	**Median**	**95% CI**
**Bacterial Adhesion Factors**	58.3 ± 4.5	58	57.1–59.5
**Anti-Phagocytic Factors**	23.4 ± 2.7	23	22.7–24.1
**Complement Proteases**	2.0 ± 0.0	2	2.0–2.0
**Exoenzymes**	2.0 ± 0.0	2	2.0–2.0
**Invasion Factors**	3.4 ± 1.2	3	3.1–3.8
**Iron Uptake Systems**	100.7 ± 5.7	100	99.2–102.2
**Magnesium Uptake Systems**	5.8 ± 1.2	5	5.4–6.1

**Phase Variation Factors**	2.0 ± 0.2	2	2.0–2.1
**Regulatory Factors**	20.2 ± 2.3	19	19.6–20.8
**Secretion Systems**	44.6 ± 5.0	43	43.3–46.0
**Serum Resistance Factors**	5.2 ± 1.1	5	4.9–5.4
**Stress Proteins**	14.1 ± 2.0	14	13.5–14.6
**Exotoxins**	25.3 ± 2.0	25	24.8–25.8
**Acridine Dyes**	6.3 ± 1.1	6	6.0–6.6
**Aminocoumarins**	9.5 ± 1.8	9	9.0–10.0
**Aminoglycosides**	11.5 ± 0.9	11	11.2–11.7
**Bicyclomycin**	1.0 ± 0.0	1	1.0–1.0
**Carbapenems**	4.5 ± 0.7	4	4.3–4.7
**Cephalosporins**	14.1 ± 1.1	14	13.8–14.4
**Cephamycins**	6.8 ± 0.9	7	6.6–7.0
**Diaminopyrimidines**	1.3 ± 0.5	1	1.1–1.4
**Elfamycins**	1.1 ± 0.4	1	1.0–1.2
**Fluoroquinolones**	21.3 ± 2.5	21	20.6–21.9
**Fosfomycins**	4.6 ± 1.0	4	4.3–4.9
**Glycopeptides**	10.4 ± 1.3	10	10.1–10.8
**Glycylcyclines**	3.4 ± 0.7	3	3.3–3.6

**Isoniazid**	2.7 ± 0.5	3	2.6–2.8
**Lincosamides**	9.7 ± 2.1	10	9.1–10.3
**Macrolides**	30.4 ± 4.4	30	29.2–31.6
**Monobactams**	2.1 ± 0.3	2	2.0–2.2
**Mupirocin**	3.1 ± 0.5	3	2.9–3.2
**Nitroimidazoles**	2.4 ± 1.143	2	2.1–2.8
**Nitrofurans**	6.4 ± 1.4	6	6.0–6.8
**Penams**	21.4 ± 1.6	21	21.0–21.9
**Penems**	0.3 ± 0.5	0	0.2–0.5
**Peptides**	11.0 ± 1.3	11	10.5–11.4
**Phenicols**	16.3 ± 1.9	16	15.7–16.8
**Pleuromutilins**	8.4 ± 1.9	8	7.9–9.0
**Rifamycins**	19.6 ± 2.0	19	19.0–20.2
**Streptogramins**	7.4 ± 1.7	7	6.9–7.8
**Sulfonamides**	1.0 ± 0.1	1	1.0–1.1
**Tetracyclines**	39.7 ± 5.8	39	38.2–41.3
**Triclosan**	5.4 ± 0.5	5	5.3–5.5

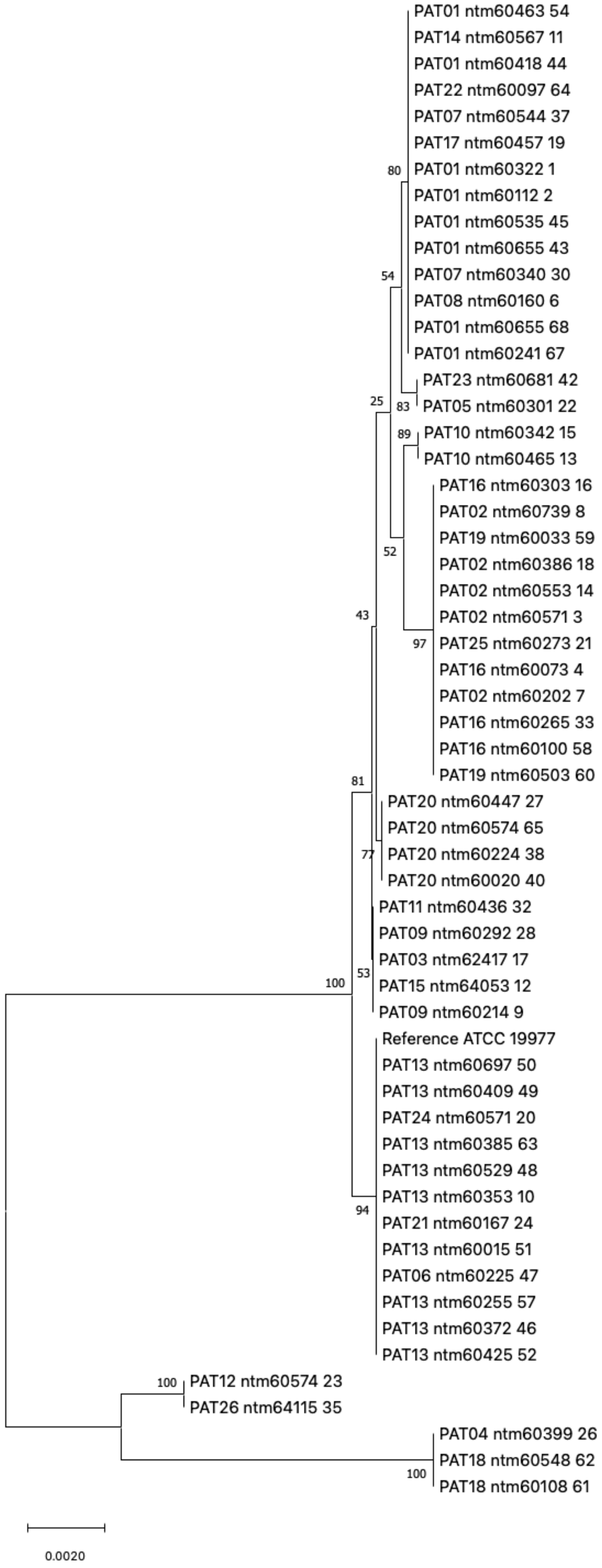

Supplementary Figure S1: Phylogenetic Tree of *M. abscessus* Strains.

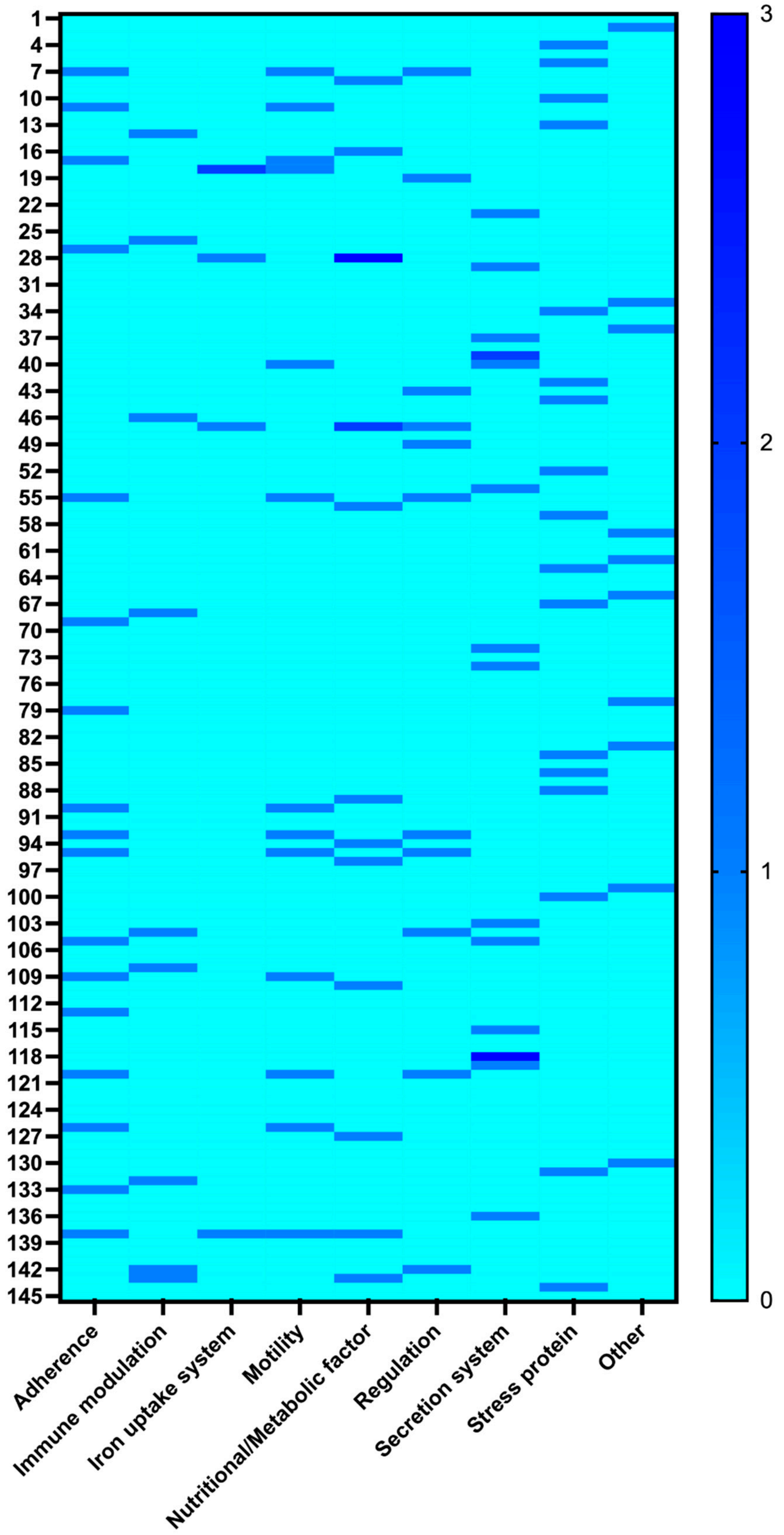

Supplementary Figures S2: Detailed Virulence Factor Distribution of Prophages.

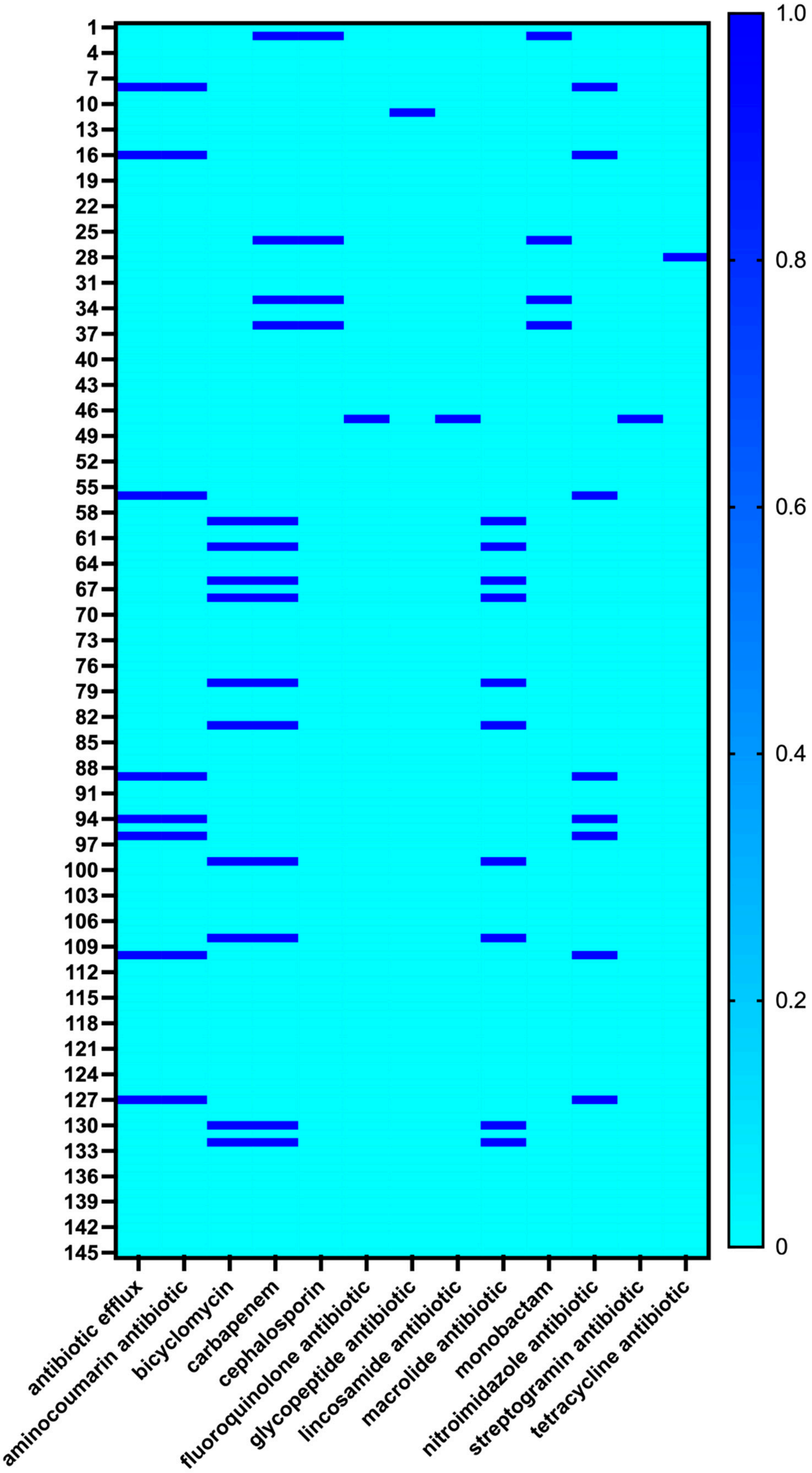

Supplementary Figures S3：Detailed Resistance Gene Distribution of Prophages.

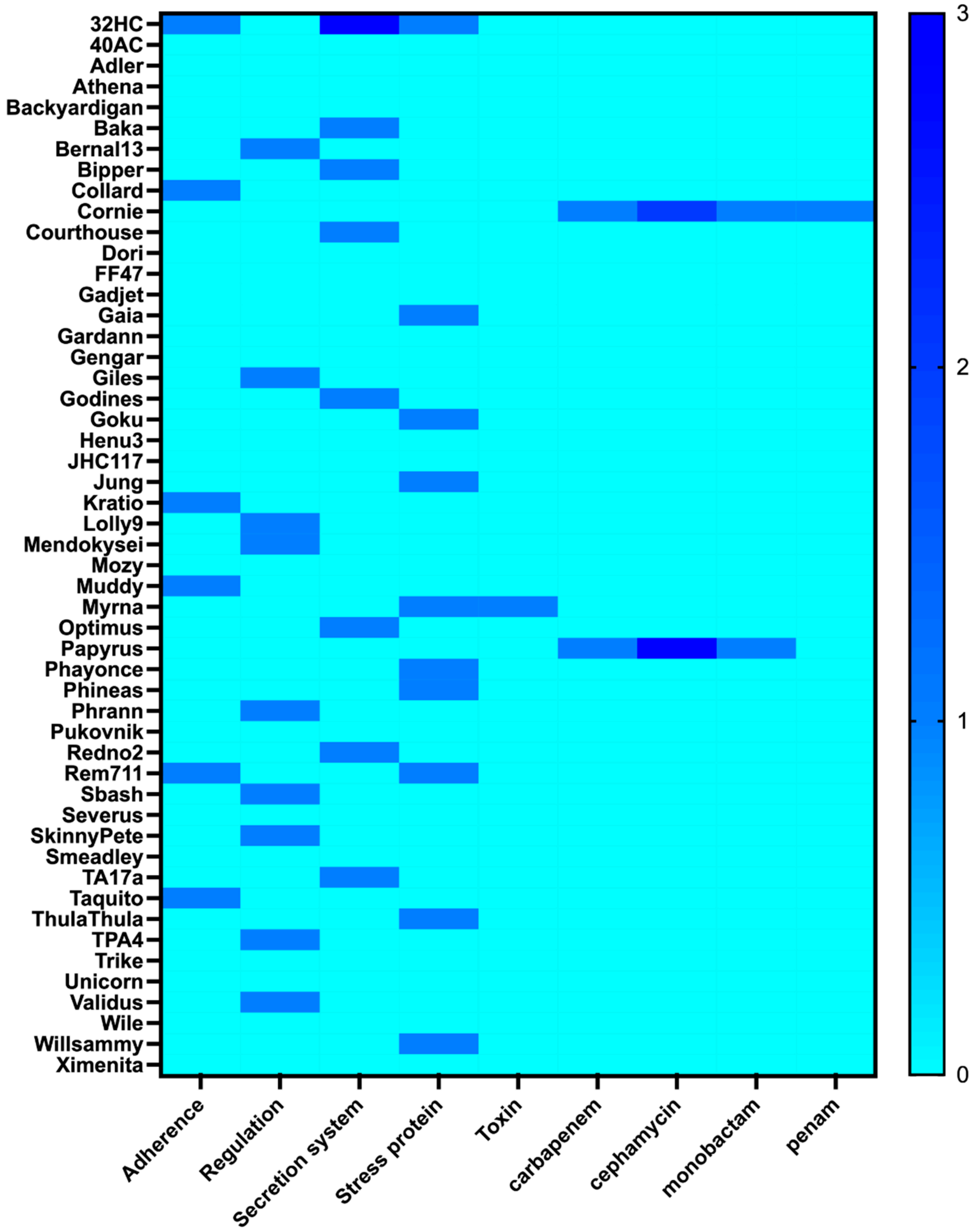

Supplementary Figures S4： Detailed Gene Distribution of Known Phages.

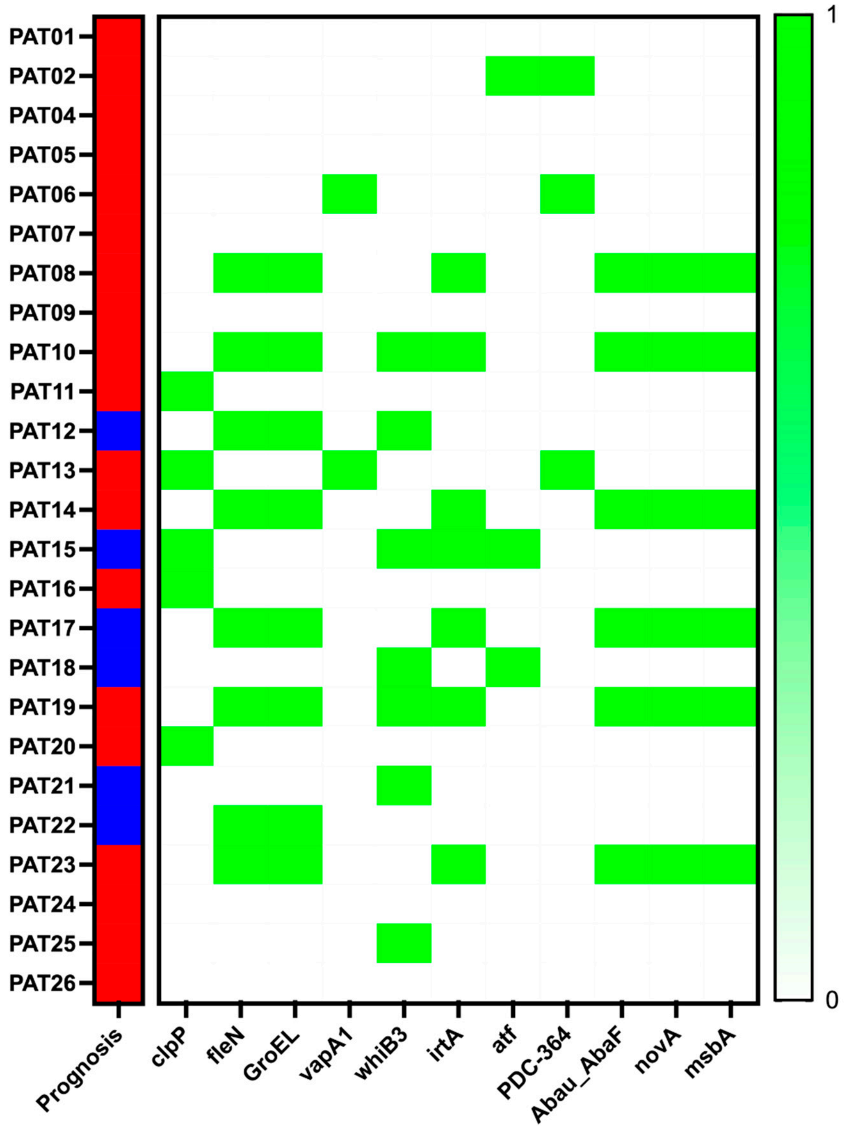

Supplementary Figures S5. Distribution of prophage-encoded virulence factors and resistance genes across patient prognosis groups.
